# Transfer of a *bla*_CTX-M-1_-carrying plasmid between different *Escherichia coli* strains within the human gut explored by whole genome sequencing analyses

**DOI:** 10.1038/s41598-017-18659-2

**Published:** 2018-01-10

**Authors:** Per Kristian Knudsen, Karianne Wiger Gammelsrud, Kristian Alfsnes, Martin Steinbakk, Tore G. Abrahamsen, Fredrik Müller, Jon Bohlin

**Affiliations:** 1 Department of Paediatric Medicine, Division of Paediatric and Adolescent Medicine, Oslo University Hospital, PB 4956 Nydalen, 0424 Oslo, Norway; 2 Institute of Clinical Medicine, Faculty of Medicine, University of Oslo, PB 1171 Blindern, 0318 Oslo, Norway; 3 Department of Microbiology, Division of Laboratory Medicine, Oslo University Hospital, PB 4956 Nydalen, 0424 Oslo, Norway; 4 Department of Molecular Biology, Domain of Infection Control and Environmental Health, Norwegian Institute of Public Health, PB 4404 Nydalen, 0403 Oslo, Norway; 5 Department of Antibiotic Resistance and Infection Prevention, Domain of Infection Control and Environmental Health, Norwegian Institute of Public Health, PB 4404 Nydalen, 0403 Oslo, Norway; 6 Department of Methodology Research and Analysis, Domain of Infection Control and Environmental Health, Norwegian Institute of Public Health, PB 4404 Nydalen, 0403 Oslo, Norway

## Abstract

Horizontal transfer of antibiotic resistance determinants contributes to dissemination of antibiotic resistance. Such transfer of resistance genes within the human gut has been documented in some *in vivo* studies. The present study investigated seven *bla*_CTX-M-1_-carrying *Escherichia coli* isolates from three consecutive faecal samples collected from one cystic fibrosis patient in a nine-months period, by analysing whole genome sequencing data. The analyses showed that the seven *E*. *coli* isolates represented three genetically diverse strains. All isolates contained *bla*_CTX-M-1_-carrying Incl1 plasmids that shared a common 101 kb backbone differing by only four SNPs. The plasmids harboured by the three different *E*. *coli* strains varied within limited regions suggestive of recombination events, according to the phylogenetic topology of the genomes of the isolates harbouring them. The findings strongly suggest that horizontal transfer of a *bla*_CTX-M-1_-carrying plasmid had occurred within the patient´s gut. The study illustrates the within-host diversity of faecally carried resistant *E*. *coli* isolates and highlights the value of collecting multiple bacterial colonies from longitudinally collected samples to assess faecal carriage of resistant enterobacteria. The clustering of the plasmids with the corresponding *E*. *coli* strains carrying them indicates that the plasmids appear to have adapted to their respective *E*. *coli* hosts.

## Introduction

During the last 10 to 15 years a global epidemic of extended-spectrum beta-lactamase (ESBL)-producing enterobacteria has emerged^1^. The CTX-M enzymes constitute by far the most common group of ESBLs^1–3^. The *bla*_CTX-M_ genes encoding the CTX-M enzymes are probably originally mobilized from the chromosomes of various species of the *Kluyvera* genus to plasmids well adapted particularly to *Escherichia coli* ^4^. Spread of CTX-M enzymes has to some extent occurred through dissemination of successful, virulent clones, e.g. *E*. *coli* ST131. According to Woerther *et al*. a huge diversity of *bla*_CTX-M_-carrying *E*. *coli* strains is present in the community with each carrier mostly harbouring his/her own strain that may disseminate in the immediate surroundings^1^. Previous studies of faecal carriage of ESBL-producing *E*. *coli* have demonstrated dissemination between household members^5,6^. Molecular characterization of the bacterial isolates revealed clonally related *E*. *coli* strains carrying the same ESBL genotype, but also genetically different strains carrying the same ESBL genotype^5^. Other recent studies have shown extensive within-host diversity in faecally carried ESBL-producing *E*. *coli* isolates with identical *bla*_CTX-M_ variants detected in genetically different *E*. *coli* isolates from the same individual^7,8^. This is suggestive of transfer of resistance genes between different strains of *E*. *coli*. The gut of humans and animals is the main reservoir of *E*. *coli* and other enterobacteria and may constitute a suitable environment for horizontal gene transfer (HGT)^9^. The large number of microorganisms, more than 10^13^ bacterial cells residing in the gut^10^, and probably also biofilm formation contribute to high bacterial density and direct contact between bacterial cells, thus facilitating HGT within the gastrointestinal tract^9,11^. A common mechanism of HGT is by conjugation with plasmids acting as vectors. Numerous studies have documented conjugational transfer of resistance genes between bacteria *in vitro* and also in some animal models^12–14^. However, not many *in vivo* studies have actually proven HGT of resistance determinants within the human gut^15–18^.

Whole genome sequencing (WGS) of bacteria offers vast opportunities for detailed characterisation of bacterial isolates, including detection and classification of plasmids^19,20^. However, assembly of plasmids from WGS data generated by high-throughput sequencing technologies that produce short reads (≈100–300 bp) might be challenging^20^. Not many studies have so far used *in silico* plasmid extraction from bacterial WGS data to study *in vivo* HGT in the human gut^21,22^.

In a study of faecal carriage of resistant enterobacteria in children^23^ we identified the plasmid-carried *bla*_CTX-M-1_ in seven different *E*. *coli* isolates from three consecutive faecal samples from one patient. The seven *E*. *coli* isolates represented three different phenotypes according to their trimethoprim-sulfamethoxazole and tetracycline susceptibility patterns. The aim of this study was to assess the genetic relationship between these seven *E*. *coli* isolates and to further characterise their *bla*_CTX-M-1_-carrying plasmids by reconstructing them *in silico* from WGS data. Specifically, we wanted to explore whether *bla*_CTX-M-1_ was located on a common plasmid in all the isolates.

## Methods

### Ethics

The study was approved by the Regional Committee for Medical and Health Research Ethics – South East (“REK sør-øst”)(reference number 581–06–03092) and the study was conducted in accordance with the regulations from the committee and the principles of the Declaration of Helsinki. Written, informed consent was obtained from the participating child´s parents.

### Collection and characteristics of the *E*. *coli* isolates and patient information

In a study of the prevalence of resistant intestinal *Enterobacteriaceae* in children^23^, one of the participants, a child with cystic fibrosis, submitted three faecal samples with time intervals of six months and three months, respectively. The faecal samples were analysed with a Direct MIC-gradient Strip Method to detect resistant *Enterobacteriaceae* ^24^. Two phenotypically different ESBL-producing *E*. *coli* isolates were detected both in sample one and sample two: one of the ESBL-producing *E*. *coli* isolates was resistant to trimethoprim-sulfamethoxazole, whereas the other was resistant to both trimethoprim-sulfamethoxazole and tetracycline. In sample three, a third ESBL-producing *E*. *coli* phenotype, susceptible to all non-beta-lactam antibiotics, was detected in addition to the two other phenotypes. For all of the three samples, one of each ESBL-producing *E*. *coli* phenotype was selected for WGS, in total seven isolates as shown in Table 1.

**Table 1:** Seven extended-spectrum beta-lactamase (ESBL)-producing *E*. *coli* isolates from three faecal samples collected from one patient at three different time points. Additional phenotypic resistance detected in each isolate is shown in the parentheses.

	Faecal samples		
	Sample 1 (time 0)	Sample 2 (after 6 months)	Sample 3 (after 9 months)
*E*. *coli* phenotypes isolated	433-tz (SXT resistant)	432-AT128 (SXT resistant)	431-Ts-o (SXT resistant)
	433-at (SXT and TET resistant)	432-CT-a-32 (SXT and TET resistant)	431-Ts-lys (SXT and TET resistant)
			431-Tz (No additional resistance)

SXT, trimethoprim-sulfamethoxazole; TET, tetracycline.

The patient had been diagnosed with cystic fibrosis at Oslo University Hospital three years prior to the first faecal sample. Her airways were chronically colonized with *Haemophilus influenzae* and *Staphylococcus aureus*. In addition, *Stenotrophomonas maltophilia* and *Achromobacter xylosoxidans*, but not *Pseudomonas aeruginosa*, had been cultured sporadically. At CF diagnosis she was treated with ceftazidime, tobramycin and cloxacillin intravenously for 14 days. After this she had not been treated with any third-generation cephalosprins or carbapenems, but she had received repeated oral antibiotic courses, mainly trimethoprim-sulfamethoxazole, but also cephalexin, cloxacillin and amoxicillin. She also received several courses with these antibiotics during the study period.

### Whole genome sequencing (WGS)

Genomic DNA was extracted using MagNa Pure 96 (Roche Diagnostics, Mannheim, Germany) according to the manufacturer’s instructions. DNA concentrations were measured using a Qubit fluorometer (Thermo Fisher Scientific, MA, USA) to determine DNA input. Isolates 431-Ts-o, 431-Ts-lys, 431-Tz, 433-tz, 433-at: Genomic libraries were prepared using KAPA HyperPlus Library Preparation Kit (Kapa Biosystems, MA, USA) and WGS was performed on an Illumina MiSeq platform using v2 reagent kits generating 2 × 250 bp paired-end reads (Illumina, San Diego, CA, USA) at the Norwegian Institute of Public Health, Oslo, Norway. Isolates 432-AT128 and 432-CT-a-32: Genomic libraries were prepared and WGS was performed on an Illumina HiSeq 2500 platform generating 2 × 125 bp paired-end reads at BGI Tech Solutions Co., Limited, Hong Kong.

### Assembly of genomes and *bla*_CTX-M-1_-carrying plasmids

**Figure 1:**
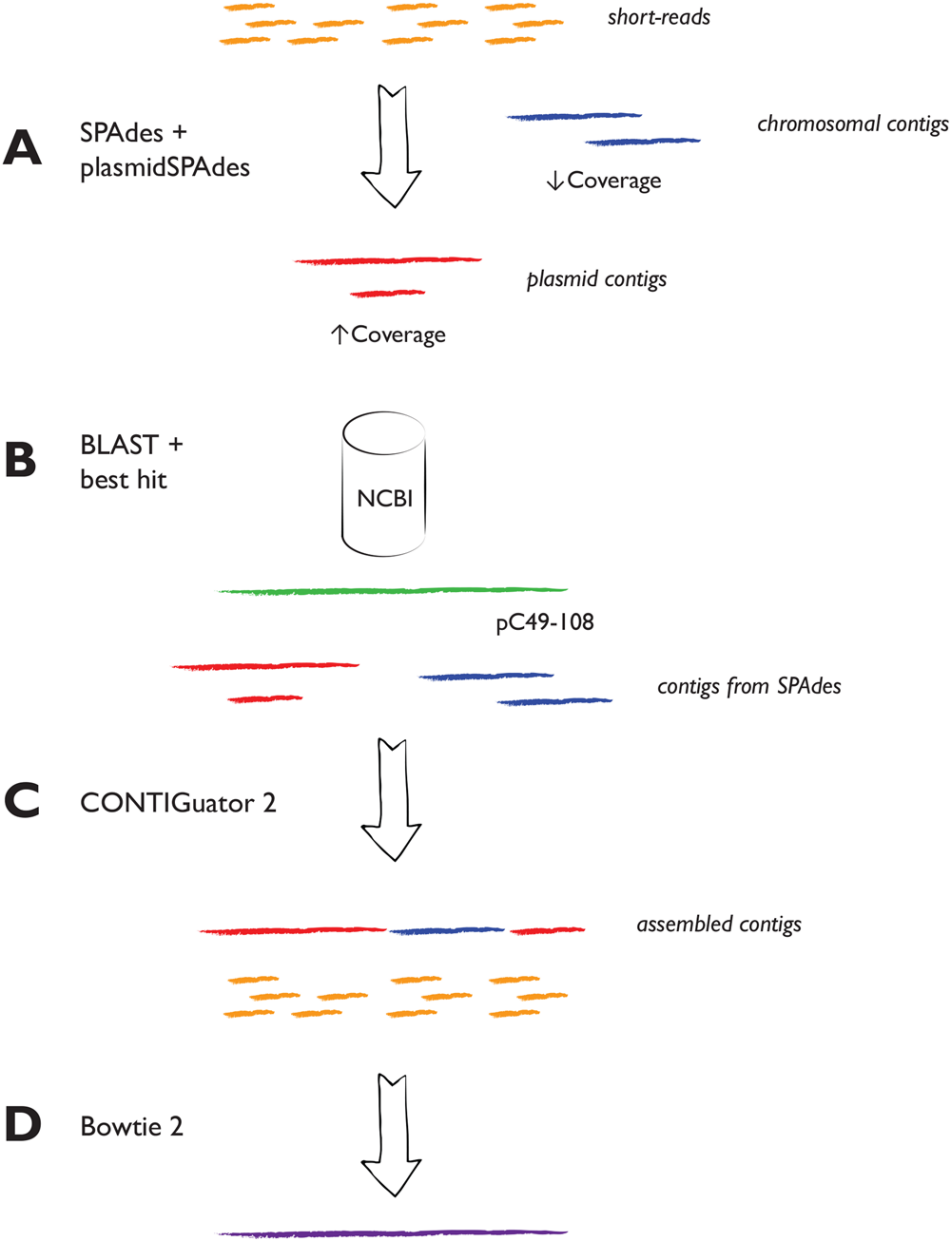
The plasmid assembly method. Short reads from each isolate are de novo assembled using SPAdes, and putative plasmid contigs are assembled and extracted by plasmidSPAdes (**A**). The putative plasmid contigs are subsequently queried against the NCBI plasmid database and if suitable matches are detected, the best hit is used as a reference sequence (**B**). The assembled plasmid sequences obtained from mapping to the reference plasmid is then used as templates for which contigs from the corresponding *de novo* assembled *E*. *coli* genomic sequences are sorted against, using CONTIGuator2 (**C**). The contigs that have a 99% nucleotide identity as well as consisting of more than 10% of reference plasmid DNA are retained as putative plasmid contigs while those falling short of the criteria are excluded. Finally, the newly assembled plasmid contigs are used as a reference to which all the original short reads from each *E*. *coli* isolate are mapped using BowTie2 (**D**).

The sequenced reads were trimmed for adapter sequences using trimmomatic^25^ and subsequently quality corrected using BayesHammer^26^. Whole genome *de novo* assembly was carried out using SPAdes v 3.9.0^27^, and PlasmidSPAdes^28^ was used to assemble and extract putative plasmid sequences from the sequenced reads (Fig. 1A). PlasmidSPAdes was run on all isolates, and the predicted plasmid contigs from the isolate having the largest contigs (isolate 431-Ts-lys), *i*.*e*. the largest contiguous predicted plasmid contigs also containing *bla*_CTX-M-1_, were BLASTed^29^ against the NCBI databases restricted to bacterial plasmids (Fig. 1B). The plasmid with the best hit (pC49–108, Accession number KJ484638) in terms of bit score and size was used as a reference for a subsequent plasmid assembly. This assembly was performed as follows: first, the plasmid-sequence of pC49–108 was used as a reference for the assembly of the putative plasmid sequences from each of the seven isolates. Second, each of the resulting assembled plasmid sequences were further used as templates of which contigs from the corresponding *de novo* assembled *E*. *coli* genomic sequences were sorted against, using CONTIGuator v 2.7^30^ (Fig. 1C). Contigs were assessed using the Artemis Comparison Tools^31^ included in the CONTIGuator package. The contigs with a 99% nucleotide identity as well as they consisted of more than 10% of reference plasmid DNA were retained as putative plasmid contigs while those falling short of the criteria were excluded. This procedure was carried out to fill in genetic regions not found in the reference plasmid. The contig-assembled plasmid was in turn used as a reference to which reads from each *E*. *coli* isolate was mapped using both BowTie2 v 2.1.0^32^ and SAMtools v 1.3.1^33^ (Fig. 1D). Assembly details of the *E*. *coli* genomes can be found in Supplementary Table S1.

### Detection of resistance genes and typing of the *E*. *coli* isolates

The assembled *E*. *coli* genomes were submitted to the web-based ResFinder service v 2.1 (Center for Genomic Epidemiology, DTU, Denmark) to identify acquired resistance genes^34^. Hits were accepted for matches with ≥99% nucleotide identity between the resistance gene in the database and the corresponding sequence in the genome, and the length of the query sequence covering ≥95% of the length of the gene in the database. Multilocus sequence typing (MLST) and serotyping of the *E*. *coli* isolates were performed *in silico* from the assembled genomes by MLST v 1.8 and SerotypeFinder v 1.1 (Center for Genomic Epidemiology, DTU, Denmark), respectively, using default settings^35,36^.

### Phylogenetic analyses of the *E*. *coli* genomes

After *de novo* assembly, phylogenetic analyses were performed on the single nucleotide polymorphisms (SNPs) from the complete *E*. *coli* genome sequences with the putative plasmid sequences removed. The plasmid sequences were excluded by mapping all reads from each *E*. *coli* genome to the *E*. *coli* K-12 strain MG1655 chromosome (Accession number NC_000913.3) with BowTie 2 v 2.1.0^32^. Further, SNP calling was carried out using parSNP v 1.2 from the Harvesttools suite^37^ by extracting the core genome, containing both coding and non-coding regions, and removal of putative recombinant regions with Gubbins v 2.2^38^. The phylogenetic analysis was performed using the RAxML maximum likelihood estimation program v 8.2.4^39^. The tree was bootstrapped 500 times and the nucleotide substitution matrix (GTR) was found with PhyML v 3.0^40^ and the R-package ‘ape’^41^ (https://www.R-project.org/). The tree was obtained using FigTree v 1.4.2 (http://tree.bio.ed.ac.uk/software/figtree/).

### Characterization and comparison of the plasmids

All putative plasmid sequences within the genome of each isolate were also identified with PlasmidFinder v 1.3 (Center for Genomic Epidemiology, DTU, Denmark)^19^ using the *Enterobacteriaceae* database with the detection threshold set to > 95% sequence identity. Characterization of the assembled *bla*_CTX-M-1_-containing plasmids by incompatibility (Inc) group and plasmid MLST (pMLST) was performed with PlasmidFinder v 1.3 and pMLST v 1.4 (Center for Genomic Epidemiology, DTU, Denmark), respectively^19^. SNP calling of the assembled *bla*_CTX-M-1_-containing plasmids was performed with BowTie2 v 2.1.0^32^ and SAMtools^33^, but the low number of SNPs (four) did not allow for a reliable phylogenetic analysis and was therefore omitted. The core backbone of the plasmids was determined with both Roary v 3.8.0^42^ (default settings) and ParSNP (fraction of coding/non-coding DNA shared amongst all plasmids). The gene presence-absence heat map of the plasmids was created with R v 3.3.3 from the Roary output and based on complete linkage hierarchical clustering with Euclidean distance. Comparison of all the assembled *bla*_CTX-M-1_-containing plasmids was performed and visualized using MAUVE^43^. All the plasmids were annotated using Prokka v 1.11^44^. Annotation of the plasmids was additionally improved by BLASTing of “hypothetical proteins” to NCBI databases.

### Data availability

The *E*. *coli* genome short reads that were generated and analysed in this work are deposited to the European Nucleotide Archive (ENA), and the accession numbers of the seven genomes are shown in Supplementary Table S1. The fasta-files containing the seven assembled plasmid sequences are also available in the Supplementary Information.

## Results

### The *E*. *coli* isolates

**Figure 2:**
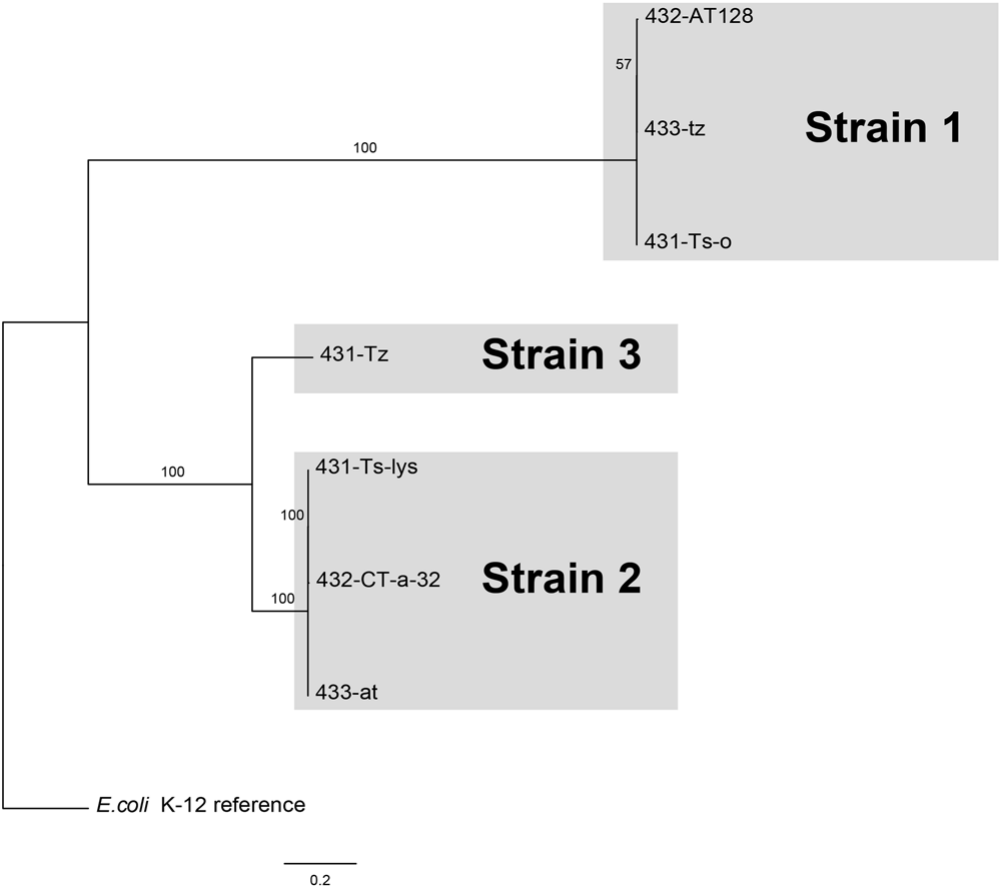
Maximum Likelihood based phylogenetic analysis of core genome SNPs from seven *bla*_CTX-M-1_-containing *E*. *coli* isolates, with the reference *E*. *coli* K-12 strain MG1655 (Accession number NC_000913.3) included as out-group. Branch numbers designate bootstrap support.

Analyses of the assembled *E*. *coli* genomes with respect to acquired resistance genes by ResFinder (Center for Genetic Epidemiology, DTU) detected *bla*_CTX-M-1_ in all seven *E*. *coli* isolates. The SNP-based phylogenetic tree presented in Fig. 2 shows that the seven isolates represent three different strains; strain 1: isolates 433-tz, 432-AT128, 431-Ts-o; strain 2: isolates 433-at, 432-CT-a-32, 431-Ts-lys; strain 3: isolate 431-Tz. The results of *in silico* typing (MLST and serotyping) of each isolate are shown in Table 2.

**Table 2:** Characteristics of seven *E*. *coli* isolates from faecal samples collected from one cystic fibrosis patient at three different time points.

Isolate-ID	Sample time	Phenotypic resistance	Strain	Sequence type (MLST)	Serotype	Resistance genes*	Plasmid replicons
433-tz	Study start	3.generation cephalosporins, SXT	1	ST1640	O124:H25	*bla*_CTX-M-1_, *dfrA14*, *dfrA17*, *sul2*	Incl1, IncFII (pRSB107), IncQ1, IncFIA, IncFIB (AP001918), Col8282, ColpVC, IncFII
432-AT128	6 months	3.generation cephalosporins, SXT	1	ST1640	O124:H25	*bla*_CTX-M-1_, *dfrA14*, *dfrA17*, *sul2*	Incl1, IncFII (pRSB107), IncQ1, IncFIA, IncFIB (AP001918), Col8282, ColpVC
431-Ts-o	9 months	3.generation cephalosporins, SXT	1	ST1640	O124:H25	*bla*_CTX-M-1_, *dfrA14*, *dfrA17*, *sul2*	Incl1, IncFII (pRSB107), IncQ1, IncFIA, IncFIB (AP001918), Col8282, ColpVC
433-at	Study start	3.generation cephalosporins, SXT, TET	2	ST6331	O82:H21	*bla*_CTX-M-1_, *dfrA17*, *sul1*, *sul2*, *tetA*	Incl1, IncFIA, Col (MG828)
432-CT-a-32	6 months	3.generation cephalosporins, SXT, TET	2	ST6331	O82:H21	*bla*_CTX-M-1_, *dfrA17*, *sul1*, *sul2*, *tetA*	Incl1, IncFIA, Col (MG828)
431-Ts-lys	9 months	3.generation cephalosporins, SXT, TET	2	ST6331	O82:H21	*bla*_CTX-M-1_, *dfrA17*, *sul1*, *sul2*, *tetA*	Incl1, IncFIA, Col (MG828)
431-tz	9 months	3.generation cephalosporins	3	ST2144	O166:H49	*bla*_CTX-M-1_	Incl1

SXT, trimethoprim-sulfamethoxazole; TET, tetracycline.

*Resistance genes detected by ResFinder (Centre for Genomic Epidemiology, DTU, Denmark) that explain the observed phenotypic resistance to 3. generation cephalosporins, trimethoprim-sulfamethoxazole and tetracycline.

### The *E*. *coli* plasmids

*In silico* analyses of the genomes from the seven *E*. *coli* isolates by PlasmidFinder detected seven different plasmid replicons in the three isolates representing strain 1: Incl1, IncFII (pRSB107), IncQ1, IncFIA, IncFIB (AP001918), Col8282, ColpVC (Table 2). This indicates that these three isolates contain several (up to seven) different plasmids. In addition, another IncFII plasmid replicon was found in one of the strain 1 isolates (isolate 433-tz). The three isolates representing strain 2 contained three plasmid replicons: Incl1, IncFIA, Col (MG828), whereas isolate 431-Tz (strain 3) contained one Incl1 replicon only (Table 2).

**Figure 3:**
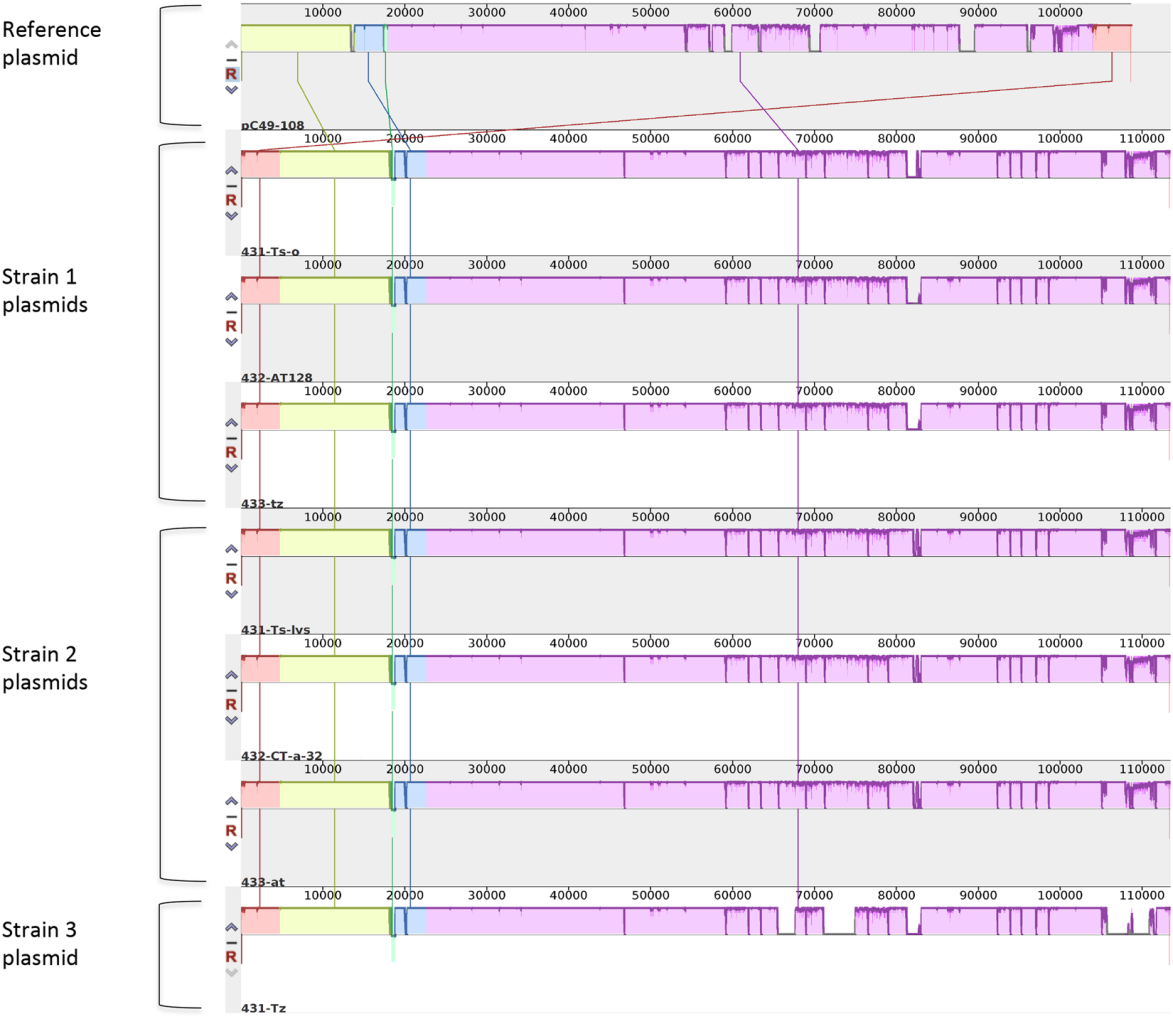
A Mauve comparison of seven *bla*_CTX-M-1_-containing Incl1/ST3 plasmids extracted *in silico* from seven *E*. *coli* isolates representing three different strains, and a reference plasmid (pC49–108, accession no. KJ484638). The height of the coloured segments indicates the degree of similarity between the plasmids where low height/missing region designates low similarity or absence of sequence. The label of each plasmid is located below the corresponding plasmid sequence.

All the assembled *bla*_CTX-M_ carrying plasmids from the seven *E*. *coli* isolates were Incl1 plasmids representing sequence type ST3. A Mauve comparison indicates that all the assembled plasmid sequences were almost identical, but with some differences among them located in delimited variable regions (Fig. 3).

All seven Incl1 plasmids shared a common 101 kb nucleotide sequence, which constitutes a core backbone of the complete 115 kb plasmid sequence (88% coverage). A corresponding SNP analysis based on the plasmid sequences (including the reference pC49–108) resulted in only four SNP differences within the backbone. Genes associated with conjugation (e.g. *traC* and *pil* genes) were detected within this common backbone. A list of annotated genes found in each plasmid is shown in Supplementary Table S2.

Comparison of the annotated plasmids with respect to the variable regions (nucleotide sequences not shared by all the plasmids) showed that these regions contained different genes, including genes encoding transposases and integrases. All three plasmids from the isolates belonging to strain 2 (the most complete plasmids) contained genes encoding TnpA transposase, transposase for Tn21 transposon, TnpR resolvase, and an integrase/recombinase (Supplementary Table S2). Corresponding genes were not found in the four plasmids from the other two *E*. *coli* strains (strain 1 and 3) that contained larger deletion blocks (Fig. 3).

**Figure 4:**
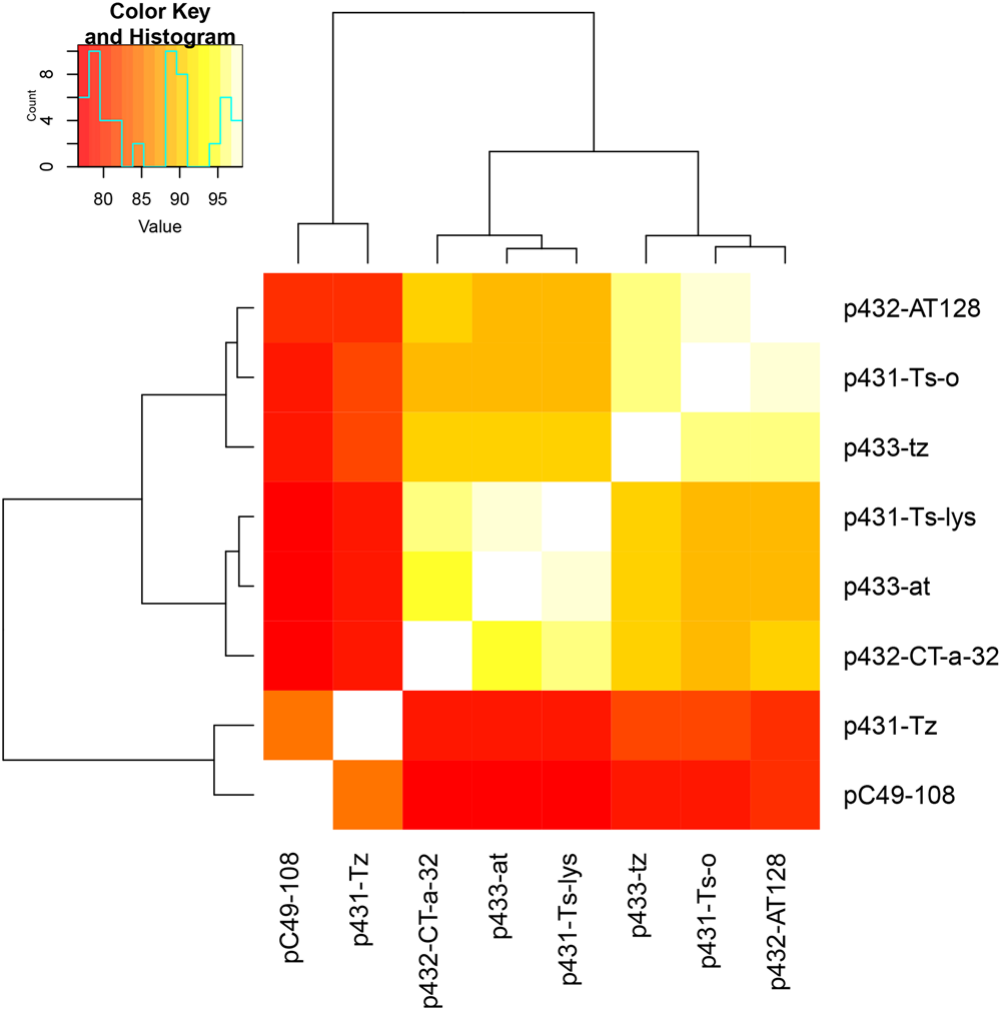
Gene presence-absence heat map of seven *bla*_CTX-M-1_-carrying Incl1/ST3 plasmids extracted from seven *E*. *coli* isolates and a reference plasmid (pC49–108, accession number KJ484638) obtained from the NCBI database. The key legend top left designates % of the genes common to the plasmids, exempting core backbone genes present in all.

Due to the close nucleotide similarity between the plasmids we created a gene presence- absence heat map of all plasmids in the present study, including the pC49–108 reference sequence, consisting of genes not included in the core backbone. From Fig. 4 it can be seen that the plasmids from *E*. *coli* strains 1 and 2 cluster according to their corresponding strain, while the plasmid from strain 3 clusters closely with the GenBank reference plasmid.

A further interrogation of the plasmids available in public repositories that resemble those in the present study the most, indicates that pC49–108, and to a lesser degree pH2291–112 (Accession number KJ484629), pC59–112 (Accession number KJ484637), pC60–108 (Accession number KJ484635) are the closest. These are all Incl1/ST3 plasmids harbouring *bla*_CTX-M-1_ detected in *E*. *coli* isolates from humans and chicken^45^.

ResFinder detected no other resistance genes in any of the *bla*_CTX-M-1_ containing Incl1 plasmids from the seven isolates. This is in accordance with the phenotypic resistance profile of the *E*. *coli* isolate 431-Tz (susceptible to all the non-beta-lactam antibiotics tested), which harboured no other plasmid replicons than Incl1 (Table 2). *dfrA* genes in combination with *sul* genes were detected by ResFinder in all the six isolates representing strain 1 and strain 2 that were resistant to trimethoprim-sulfamethoxazole. *tet* genes were detected in the three tetracycline resistant strain 2 isolates (Table 2). These genes are probably located on some of the other plasmids detected in these strains (Table 2).

## Discussion

In this study we explored seven ESBL-producing *E*. *coli* isolates and their corresponding *bla*_CTX-M-1_-carrying plasmids isolated from faecal samples from one cystic fibrosis patient at three different time points. The assessment of the isolates and the plasmids was exclusively based on WGS analyses. Assembly of plasmids from WGS short reads is challenging^20^, and complete reconstruction is often not possible due to the presence of repeat sequences, especially in large plasmids ( > 50 kbp)^46^. Here we aimed to reconstruct plasmid sequences by combining *de novo* assembly and reference-based read mapping relying on the successful detection of a reference plasmid (pC49–108) in the NCBI databases with a high degree of similarity with our plasmid sequences.

All seven ESBL-producing *E*. *coli* isolates carried *bla*_CTX-M-1_, but the isolates represented three different sequence types and serotypes. The phylogenetic analyses (Fig. 2) confirmed that this was in accordance with three genetically diverse groups of isolates that we designated strain 1, 2 and 3 (Table 2).

All seven *bla*_CTX-M-1_-carrying plasmids belonged to the same Inc group (Incl1) and represented the same pMLST (ST3). They shared a common 101 kb nucleotide core backbone containing only four SNP differences. This high degree of similarity between the plasmid backbones, also visualized by the Mauve comparison in Fig. 3, strongly suggests that they originate from a common plasmid that may have been transferred between the different *E*. *coli* strains. The environment within the human gut is favourable for horizontal gene transfer^9,47^, and the transfer of the *bla*_CTX-M-1_-containing plasmid is likely to have happened within the patient’s gut. It is well known that plasmids belonging to the Incl1 group often carry ESBL-coding genes and that they have the capacity of conjugational transfer^48^. Genes associated with conjugation (e.g. *traC* and *pil* genes, shown in Supplementary Table S2) were detected within the common backbone of all our plasmids and support the hypothesis that the plasmids probably have been transferred between different *E*. *coli* strains within the patient´s gut. The diversity of CTX-M-1 producing *E*. *coli* isolates found in our study is similar to findings in recent studies that have demonstrated great within-host diversity in faecally carried ESBL-producing *E*. *coli* isolates^7,8^. Stoesser *et al*. sequenced the genomes of 16 *E*. *coli* colonies from each of eight faecal samples from different individuals in Cambodia^7^. In two of the eight individuals, the same *bla*_CTX-M_ variant occurred in different *E*. *coli* clones, and/or different *bla*_CTX-M_ variants occurred in the same clone. Similar findings were demonstrated by Jørgensen *et al*. in a longitudinal study of faecal carriage of ESBL-producing *E*. *coli* and *Klebsiella pneumoniae* ^8^; ESBL production commonly occurred in diverse strains within the same host, and the same *bla*_CTX-M_ variant was detected in genetically different *E*. *coli* isolates from the same faecal sample. These findings suggest horizontal transfer of *bla*_CTX-M_ within the gut, but none of these studies assessed the mobile genetic elements (e.g. plasmids) that may have been involved in such transmission. Conlan *et al*. studied transmission of plasmids by analysing WGS data obtained from several sequencing platforms. They detected *bla*_KPC_-carrying pKpQIL plasmids with a common backbone in several *K*. *pneumoniae* isolates representing two different STs and in an *E*. *coli* isolate from faecal samples collected from the same patient at different time points^21^. Horizontal transfer of resistance genes may be facilitated by antibiotic exposure^49^, and the extensive antibiotic exposure in our patient may have promoted transfer of *bla*_CTX-M_ between different *E*. *coli* strains. Such antibiotic treatment may also select resistant (e.g. ESBL-producing) strains of enterobacteria and concomitantly supress dominant populations of anaerobic species in the gut, thus promoting cell-to-cell contact between enterobacteria that enhances HGT. Stecher *et al*. showed that gut inflammation could boost HGT between pathogenic and commensal *Enterobacteriaceae* ^50^. This may also be of relevance in patients with CF since this disease is associated with intestinal inflammation^51–53^.

The *bla*_CTX-M-1_-carrying plasmids contained variable regions with genes encoding transposases and integrases (Supplementary Table S2). Notably, the most complete plasmids, harboured by the strain 2 isolates, contained several genes associated with recombination and transposable elements that were not detected in the plasmids from strains 1 and 3. The plasmids from *E*. *coli* strains 1 and 3 contained deletion blocks (Fig. 3) that may indicate that recombination events have happened in the plasmids within the different hosts. The phylogenetic SNP analysis of the *E*. *coli* genomes (Fig. 2), without the putative plasmid sequences, indicates that the plasmids differ according to the phylogenetic topology of the genomes of the isolates harbouring them. The gene presence-absence heat map of the plasmids (Fig. 4) also show that the plasmids cluster according to the three different *E*. *coli* strains, suggesting that the plasmids may have adapted within their respective hosts. The differences between the groups of clustered plasmids shown in Fig. 4 are most likely due to deleted and/or acquired accessory genes.

The closer clustering of the plasmids discussed above, as compared to the pC49–108 reference, and the large shared backbone of all plasmids considered, may suggest what best can be described as an adaptive ability. It has been demonstrated that plasmids co-evolve with their hosts^54,55^, which may result in the loss of genes conferring fitness cost. In this respect it is interesting to note that the strain represented by the single 431-Tz isolate, that contained the plasmid with largest deletion blocks, was isolated at a later time point than the isolates representing the other strains. It is therefore tempting to speculate that the plasmid was introduced to one of these strains and subsequently transferred to the other strains where co-evolution with the respective host in each strain has resulted in deletion events in the plasmids. The heavy antibiotic exposure to the patient may also have promoted the adaptive events in the plasmids within the *E*. *coli* hosts, as described by Porse *et al*.^54^. In an experimental study they demonstrated adaptive evolution by deletion of a costly region from a multidrug resistant plasmid in *E*. *coli* during antibiotic exposure, and that the increased plasmid stability was maintained also after the antibiotic selection pressure was removed.

One limitation of the present study is the use of short read sequencing data to reconstruct large plasmids (>100 kb). Several investigators have demonstrated that it may be challenging to obtain correct assemblies of large plasmids from short reads^46,56,57^. The occurrence of multiple repetitive nucleotide sequences (e.g. transposons and IS-sequences) and/or recombination events, both known to be common in plasmids, are demanding obstacles to overcome. For instance, if resistance genes are flanked by repetitive sequences (e.g. transposon elements) that are longer than the read lengths obtained by short-read based sequencing, it can be difficult to determine the correct genetic context of the resistance genes^57^. Therefore, long-read based sequencing methodology (e.g. Single Molecule Real-time Sequencing (SMRT), Pacific Biosciences or Nanopore sequencing, Oxford Nanopore) may hold great promise in obtaining properly assembled and closed plasmids^20^. Without these methods available, and only relying on the short read sequencing approach used in the present study, there may be inconsistencies regarding the plasmid’s variable regions as well as reverse complemented regions, and the extracted plasmids cannot be considered as fully closed. However, all the plasmids in our study cluster according to the *E*. *coli* strain to which they belong (Fig. 4), although the cluster analysis is based only on genes from the plasmid’s variable regions. There may be a possibility that the plasmids extracted by our method are divided between multiple plasmids, which may appear as one contig from the assembled short read data, or that the plasmid backbone has been integrated into different plasmid segments. However, such segments would most likely have been detected during the contig extraction step (Fig. 1C) by inconsistencies between the plasmids of the multiple isolates. We acknowledge that this could be difficult to verify in studies including only single isolates. The consistency of the finding of *bla*_CTX-M-1_-carrying Incl1 plasmids sharing a common backbone in two different *E*. *coli* strains (strain 1 and 2) isolated from three consecutive faecal samples during a nine-months period, strongly supports that a common plasmid is present in the different strains. As Incl1 plasmids are known to have conjugational capacity^48^, it is not unlikely that the plasmid has been horizontally transferred between the two strains and even to or from a third strain. However, this cannot be proven due to the methodological limitations described. While the availability of long read sequencing technology is increasing, short read sequencing platforms are still far more commonly available. Thus, extraction of plasmids from short-read data, and methods facilitating this, will probably still be used for some time to come.

In summary, we reconstructed *bla*_CTX-M-1_ containing Incl1 plasmids *in silico* from *E*. *coli* whole genome sequenced short reads by combining *de novo* assembly and reference-based read mapping relying of the successful detection of a reference plasmid in the NCBI databases with a high degree of similarity with our plasmid sequences. Seven *bla*_CTX-M-1_-carrying plasmids extracted from isolates that represented three genetically diverse  *E*. *coli* strains isolated from faecal samples from one individual shared a common backbone. The findings strongly suggest horizontal transfer of the *bla*_CTX-M-1_-carrying plasmid between different *E*. *coli* strains within the patient´s gut, although our results need to be interpreted with some caution due to the limitations associated with plasmid assembly from short-read sequencing data. The differences between the plasmids, indicative of recombination events, corresponded to the *E*. *coli* strain carrying them, indicating that the plasmids appear to have adapted to their respective *E*. *coli* hosts.

The study illustrates the within-host diversity of faecally carried resistant *E*. *coli* isolates and highlights the value of collecting several bacterial colonies from longitudinally collected samples to assess faecal carriage of resistant enterobacteria.

## Electronic supplementary material

Supplementary Tables S1 and S2

Plasmid sequence p431-Ts-lys

Plasmids sequence p431-Ts-o

Plasmid sequence p431-Tz

Plasmid sequence p432-AT128

Plasmid sequence p432-CT-a-32

Plasmids sequence p433-at

Plasmids sequence p433-tz
